# Muscle Growth and Poultry Meat Quality Issues

**DOI:** 10.3390/nu4010001

**Published:** 2011-12-22

**Authors:** Massimiliano Petracci, Claudio Cavani

**Affiliations:** Department of Food Science, University of Bologna, 47522 Cesena (FC), Italy; Email: claudio.cavani@unibo.it

**Keywords:** poultry, muscle growth, meat quality

## Abstract

Over the past 50 years the worldwide growing demand of poultry meat has resulted in pressure on breeders, nutritionists and growers to increase the growth rate of birds, feed efficiency, size of breast muscle and reduction in abdominal fatness. Moreover, the shift toward further processed products has emphasized the necessity for higher standards in poultry meat to improve sensory characteristics and functional properties. It is believed that genetic progress has put more stress on the growing bird and it has resulted in histological and biochemical modifications of the muscle tissue by impairing some meat quality traits. The most current poultry meat quality concerns are associated with deep pectoral muscle disease and white striping which impair product appearance, and increased occurrence of problems related with the meat’s poor ability to hold water during processing and storage (PSE-like condition) as well as poor toughness and cohesiveness related to immaturity of intramuscular connective tissue. This paper is aimed at making a general statement of recent studies focusing on the relationship between muscle growth and meat quality issues in poultry.

## 1. Introduction

Over the past few years, meat production and market have undergone several negative events that have impaired the image of this essential food product from the consumer’s standpoint [1]. The image of meat and meat products is relatively negative due to their content in fat and saturated fatty acids, cholesterol, sodium and any other substances (e.g., nitrosamines) that somehow can be involved in most prevalent diseases of Western societies like cardiovascular diseases and diabetes mellitus [2] and cancer [3,4,5]. In fact, epidemiological data suggests a relationship between meat consumption or dietary heme and risk of colon cancer [3,6]. Reduction in meat consumption has been accentuated by a series of scandals and animal health problems which have hit livestock production, such as BSE, dioxins in meat and avian influenza. Within this context, the poultry meat has maintained its identity and a higher value compared to other species for several reasons. Indeed, worldwide poultry meat production and consumption have increased rapidly and, in many parts of the world, per capita consumption of poultry meat will continue to grow [7]. Relatively low and competitive prices compared to other meats, the absence of cultural or religious obstacles, and dietary and nutritional properties are the main factors explaining poultry meat’s attractiveness [8]. 

Regarding nutritional aspects, poultry meat well fit the current consumer demand for a low-fat meat with a high unsaturation degree of fatty acids and low sodium and cholesterol levels. Poultry meat may also be considered as “functional foods”, which provide bioactive substances with favorable effects on human health, like conjugated linoleic acid (CLA), vitamins and antioxidants, and a balanced *n*-6 to *n*-3 PUFA ratio [9,10]. It should also mentioned that the changes in consumer’s lifestyle in developed countries have led to a meat market more and more addressed towards easy-handled and processed products (“convenience food”). This trend has been exploited since long time by the poultry industry, which made strong investments in the processing area, by increasing the availability of poultry in a large variety of processed ready meals [7]. 

This growing demand for poultry meat has resulted in pressure on breeders, nutritionists and growers to increase the growth rate of birds, feed efficiency, size of breast muscle and reduction in abdominal fatness. Today, chickens and turkeys are marketed in about half the time and at about twice the body weight compared to 50 years ago [11]. These improvements are mainly due to the high heritabilities of body weight and body composition during breeding [12]. This kind of selection has obviously put more stress on the growing bird and some believe it has resulted in histological and biochemical modifications of the muscle tissue [11]. Several studies evidenced that fast growing strains exhibit a high incidence of spontaneous or idiopathic myopathies (e.g., deep pectoral muscle disease) and an increased susceptibility to stress-induced myopathies which may have great implications for meat quality and incidence of abnormal conditions such as pale, soft and exudative (PSE)-like meat [13,14,15,16]. Moreover it is also believed that selection for muscle growth has resulted in an increase in meat quality problems associated with toughness and poor cohesiveness, color, and water holding properties [17]. It should also be recognized that, when selling parts or deboned meat, meat quality issues such as water holding capacity, appearance, and texture became the responsibility of the processors and as consequence meat quality has become more economically important [18].

## 2. Muscle Myopathies

**Figure 1:**
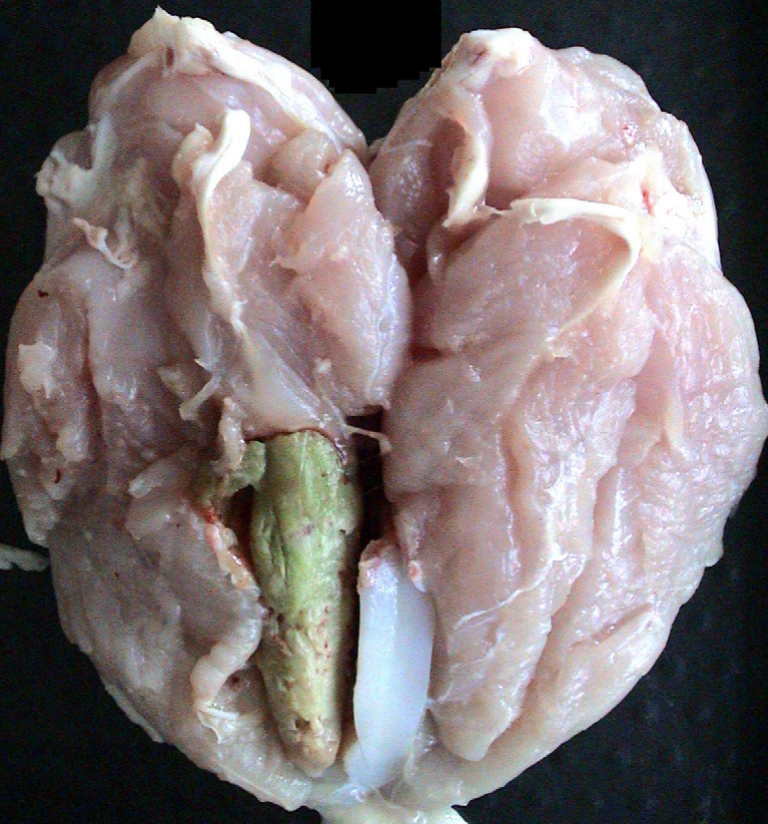
Deep pectoral myopathy [27]. With permission from Poultry Science Association.

With the increase in growth rate and muscle size, there has been an increase in incidence of pectoral myopathies (e.g., focal myopathy) [17]. Among these, deep pectoral disease (DPM) has the more important impact on final product quality issues (Figure 1). Deep pectoral disease, also known as Oregon disease or green muscle disease, was first described in 1968 as “degenerative myopathy” in turkeys [19] and it was subsequently studied at the Oregon State University [20,21]. Even though this condition was first recognized in adult meat-type turkey and chicken breeders, it has become more and more common in meat-type growing birds [22,23,24]. DPM occurs exclusively in birds that have been selected for breast muscle development [21]. It is generally recognized that deep pectoral myopathy is an ischemic necrosis that develops in the deep pectoral muscle (*supracoracoideus* or *pectoralis minor* muscle) mainly because this muscle is surrounded by inelastic fascia and the sternum, which do not allow the muscle mass to swell in response to the physiological changes occurring when muscles are exercised, as in wing flapping [25]. It has been estimated that, in turkeys and broilers, the supracoracoid increases in weight by about 20% during activity for the huge blood flow into the muscle. The increased size of the muscle is so marked in the heavy breeds that the muscle becomes strangulated and ischemic, because the increased pressure within the muscle occludes the blood vessels and causes a necrosis of the muscle The lesion does not impair the general health of birds and is generally found during cut-up and deboning; moreover, it can be both unilateral or bilateral, affecting just one or both *pectoralis minor* muscles, respectively. No public health significance is associated to deep pectoral myopathy, but it is aesthetically undesirable. The fillet should be removed whereas the rest of the carcass is still fit for human consumption. However, the required trimming operations cause the downgrading of the products and produce an economic loss for the industry, especially because it affects the more valuable part of the carcass. The incidence of carcasses affected by deep pectoral myopathy was estimated to be just below 1% [26]. The incidence of DPM increases with market weight in broilers, with more cases reported in higher-yielding strains and in males. Increased bird activity (flock nervousness, flightiness, struggle, and wing flapping) induced by factors such as feed or water outages, lighting programs and intensity, human activity, and excessive noises in and around chicken houses should be looked at as a trigger for the development of DPM in broilers [27]. 

Based on data obtained by Bianchi *et al*. [26], DPM prevalence can be different when diverse breeds are considered, suggesting that genetics may play an important role in the determination of this condition. As a consequence, genetic selection against DPM has been undertaken by broiler breeding companies. Moreover, recent developments in whole-genome selection using dense DNA-markers should provide effective and powerful tools to reduce DPM importance in the future. 

## 3. Meat Quality Abnormalities

Increased concerns related to meat quality issues are prevalent. As previously reported, this is not surprising since the move towards further processing has led to increased handling of the product; thus, creating new appearance issues that were not apparent in a whole bird or cut-up market. In addition the age at which slaughter of poultry occurs has been continually declining. Therefore, muscle color and texture attributes must be monitored in order to determine whether an age or genetic components exists [11]. 

### 3.1. PSE-like Breast Meat

**Figure 2:**
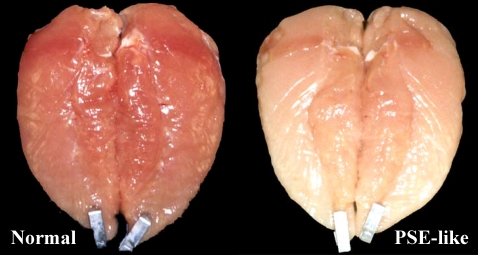
Pale, soft and exudative (PSE)-like broiler breast meat.

One of the most frequent challenges to the meat industry associated with the intensive selection for increased muscling is the development of pale, soft and exudative (PSE) meat. The term PSE was originally a descriptor for a pork product, characterized by light color, flaccid texture, poor water-holding capacity and substantially reduced cooking yield. In swine, a genetic single mutation in the ryanodine receptor of the sarcoplasmatic reticulum involved in calcium release has been identified and has been associated with animals that are stress-susceptible and prone to developing PSE meat [11,28]. With the advent of technologies to identify and eliminate this major cause of extreme cases of PSE, a great reduction in the incidence and severity of PSE has been realized, even if products with poor water holding capacity still exist [11]. The suggestion that a pale, soft, and exudative (PSE-like) condition exists in poultry was mentioned some decades ago (Figure 2). However, to date, there is no evidence to support or refute a genetic mutation in chicken and turkeys as related to PSE development [16].

It is generally accepted that the rate of *post mortem* metabolism is the major contributor to the variation in fresh meat quality and processing functionality of meat proteins. This loss of product and protein quality is attributed to protein denaturation caused by a combination of acid conditions along with high muscle temperature in very early *post mortem* muscle (within 30 min after the death of the animal). The flight (breast) muscle of chickens and turkeys are entirely Type-IIb fibers (glycolytic), capable of short bursts of activity for the “Fight or flight” response. Energy is produced anaerobically via glycolysis, whereby glycogen is broken down to lactic acid, which is normally removed by the blood. The metabolism of the breast muscle and conditions at slaughter could contribute to PSE-type meat because there are large glycogen stores within the breast muscle with a high propensity to produce lactic acid (entirely glycolytic) and therefore the potential for rapid drop in pH and/or low ultimate pH *post mortem* exists. In addition, there is the potential for high muscle temperatures due to flapping, struggle, stress, and high metabolic rate in the lead-up to slaughter and the large breast muscle mass (particularly in turkeys) being difficult to chill *post mortem* [11]. 

Several studies have been conducted to establish directly or indirectly the main causes of PSE-like condition in poultry [15,29]. These studies can be divided into two categories: those evaluating the role exerted by genetic selection and those concerning the effect of environmental factors. As for genetics, it has been shown that selection for body weight or muscle development has induced histological and biochemical modifications of the muscle tissue, which can be related with PSE-like condition [11]. Numerous studies conducted evidenced that modern rapidly growing strains of meat poultry exhibited an elevated incidence of spontaneous or idiopathic myopathy and an increased susceptibility to stress-induced myopathy [30,31]. These pathologies are attributable to alterations in intracellular calcium homeostasis [31,32] and consequent changes in sarcolemmal integrity and may result from excessive myofiber hypertrophy and inadequate development of support tissues and vascular supply [33,34]. These authors stated that these myopathies may have profound implications for meat quality and the incidence of specific conditions such as PSE-like meat. It should also be mentioned that some recent studies did not identify any antagonism between growth rate or muscle development and breast meat quality parameters such as water retention and processing ability [14]. In chickens, Berri *et al*. [35] suggested that selection for increased muscle yields and against fat deposition could exert cumulative effects on muscle metabolism, decreasing glycogen storage and thereby reducing the extent of *post mortem* acidification. As a consequence of higher ultimate pH, the WHC and processing ability of the meat was improved. Also in turkeys, Werner *et al*. [36] stated that greater body weight and the larger muscle fibers of the fast growing strains had no negative impact on the *post mortem* muscle-to-meat transition process and the incidence of degenerated fibers or of haemorrhages.

Among environmental factors to induce PSE-like meat occurrence, heat stress during the end of the growing phase or preslaughter period seems to play the major role [37]. Faster growing or heavier birds have been shown to be more susceptible to heat stress indicated by great metabolic heat production, increased body temperature, and mortality. Sandercock *et al*. [31,38] found that rapidly growing lines of birds may exhibit a reduced thermoregulatory capacity compared with their genetic predecessors and may thus be more susceptible to heat stress during the preslaughter period and to consequent problems including muscle damage, acid-base disturbances, and reduced meat quality. Acute heat stress has been demonstrated to increase superoxide free radical production in chicken skeletal muscle [39]. This mechanism may be responsible for the transport stress- and heat stress-induced muscle damage and for the changes in muscle and meat quality observed in broilers. Thus muscle cell metabolism and alterations in sarcolemmal integrity and tissue structure associated with oxidative damage and myopathy may have profound implications for meat quality and the incidence of specific conditions such as PSE-like meat.

Today, with the advent of “omics” science there are more possibilities to further investigate these problems. In contrast to genomics, proteomic studies are becoming more and more popular to study the relationship between genome and functional properties of meat. While genome contains information on which genes and alles are present in the genome, the proteome contains information on which genes are actually being expressed and translated into proteins. Thus, understanding the variations and different components of proteome with regard to certain quality or processing parameters will lead to knowledge that can be used in optimizing the conversion of muscles to meat [40]. *Post mortem* proteomic analyses provide important molecular information on related metabolic pathways and help to identify mechanisms underlying muscle conversion to meat and meat quality development [28]. Some preliminary studies carried out on PSE-like meats using proteomic tools indicated that the process of muscle to meat conversion was probably modified in muscles having predominant fast glycolitic metabolism (e.g., *pectoralis major* and *pectoralis minor* muscles) and this could cause modifications in proteolytic enzyme functionalities and/or muscle protein denaturations. In addition, the possible modifications of two glycolytic enzymes (fructose biphosphate aldolase and glyceraldehyde 3-phosphate dehydrogenase) could explain the differences in the rate of pH decline between normal and PSE-like breast meat [41]. Complementary, proteomic analyses could be used to try to detect, as early as possible, animals carrying these modifications which can be supposed to be prone to produce meat with poor quality [41]. 

Recently, there are also few studies about understanding how dietary nutrients can impact on gene expression (nutrigenomics) focused on meat quality issues in poultry. It is well known that supranutritional dietary levels of tocopherols had beneficial effects to delay the initiation of oxidation and loss of quality in poultry and this can effectively inhibiting the development of PSE-like meat hence improving meat functional properties [42]. This is because the integrity of the cell membrane is thought to influence liquid losses and protection of membranal lipids against lipid oxidation by endogenous vitamin E has been suggested to be the mechanism responsible for the positive influence of dietary vitamin E on the water holding capacity [43]. Li *et al*. [44] using a nutrigenomic approach found that long-term dietary vitamin E supplementation leads to altered transcription of genes related to lipid metabolism via major signal transduction pathways involving some specific enzymes (protein kinase C and phosphatidylinositol 3-kinase), thereby potentially improving fatty acid synthesis and composition of body fat. These authors concluded that vitamin E beneficial effects on lipid stability in muscle and meat quality and fatty acid composition was probably also due to its influence on the expression of genes related to lipid metabolism.

### 3.2. Intramuscular Connective Tissue Defects

A newer emerging quality issue in poultry is the poor cohesiveness of meat due to immaturity of intramuscular connective tissue (IMCT) in relation to the very early slaughter age of modern chicken and turkey strains. The structural integrity of muscle fibers is maintained by three layers of IMCT: (i) the endomysium that surrounds individual skeletal muscle fibers; (ii) the perymisium that bundles a group of muscle fibers, and (iii) the epimysium that ensheathes the whole muscle [45]. 

IMCT is principally composed of cells and extracellular matrix, which is composed of collagen, proteoglicans and glycoproteins [46]. The epimysium is often thick and tough; however, it is usually separated from cuts of meat so it plays a minor role in determining meat quality. The IMCT is thus the combined peri- and endomysium depots, even if perimysium representing about 90% of total connective tissues in muscles [47,48]. The strength of IMCT is based on collagen fibrils and there are cross-bridges between the collagen molecule units and also between the collagen molecules. These cross-bridges determine the physical strength and heat stability of IMCT. The number and stability of cross-bridges increase with age determining a reduced tenderness. Modern poultry is not tough, but the problem is increasingly the opposite. The collagen content of lean meat is 0.2–0.4%. In fast-growing birds the collagen is immature resulting in low heat stability. Consequently, poultry meat is tender, but may turn fragile, even mushy [49]. 

**Figure 3:**
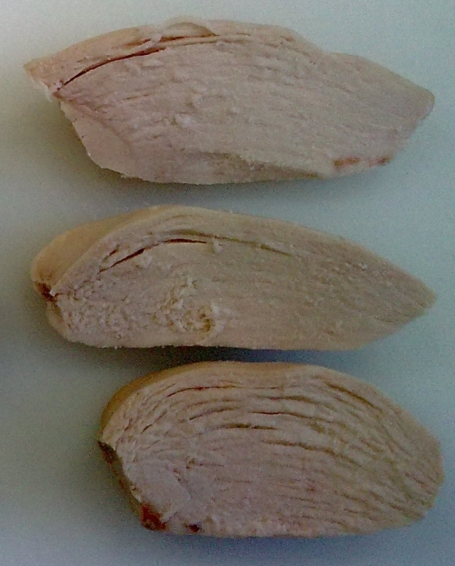
Broiler breast meat with poor cohesiveness.

Voutila *et al*. [50] indicated that currently there are two emerging types of defect in commercial poultry meat: (1) cooked chicken breast meat is generally fragmented (soft) (Figure 3); and (2) raw turkey breast meat is so loose in structure (disintegrated) that it is possible to pull the muscle fiber bundles away with the fingers. The disintegration of cooked turkey meat has been reported by Swatland [51]. The mushy structure of cooked chicken breast meat can be perceived so that the need to chew before swallowing the piece of meat is minimal [52]. 

During breast muscle development in modern turkey and broiler breeds, an increase in the cross-section of muscle fibers is greater than that in endomysial and perimysial connective tissues, and this suggests that selection for rapid growth has created muscles that outgrow their life support systems and bring about muscle damage [17]. Thus, the final trigger to the disintegration of turkey breast meat could be the formation of large intercellular spaces, as fluid that is released from myofibrils is lost from the muscle fibers *post mortem* [51]. Recently, Ahn *et al*. [53] found that endomysium and perymisium thickness of breast muscles was larger and much smaller, respectively, in fast-growing broilers if compared with slow-growing egg-type chickens. This evidence suggested that the growth of endomysium and perimysium may separately be regulated and this can later translate in poor slicing and fragmentation seen in cooked deli products. Voutila *et al*. [50] using a differential scanning calorimetry approach evidence that one feature could not be directly associated with weakening of meat structure.

**Figure 4:**
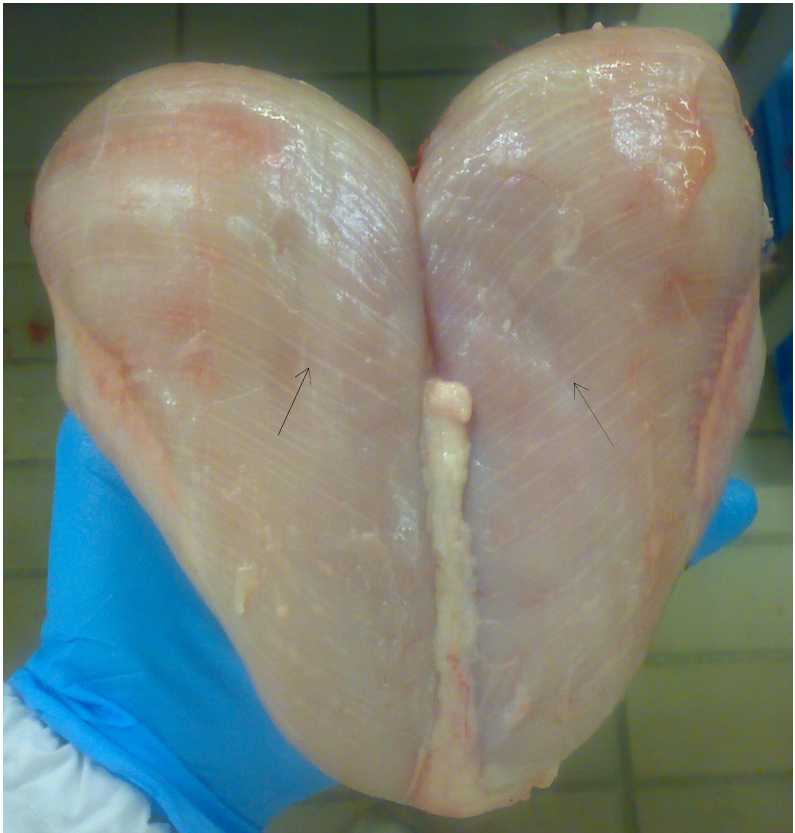
Broiler breast meat with “white striping” defect.

A very new quality issue was recently observed regarding the appearance of breast muscle. McKee [54] indicated that one of emerging meat quality problems is the appearance of white striping or striations in poultry breast fillets following the directions of muscle fibers (Figure 4). While the phenomenon has not be linked to any particular eating attributes of cooked poultry, it does affect the appearance of raw meat and would possibly lead to consumers not selecting the product due to its appearance. Histological observations indicated an increase in degenerative and atrophic fibers in breast fillets affected by white striping. 

As previously discussed for PSE-like issue, proteomics can be also very helpful to identity potential protein markers for understand defects such as poor texture and white striping in poultry related with intramuscular connective tissue development. For example, there are an increasing number of publications trying to reduce toughness problems in beef meat [55,56]. However, until now, no clear relationship between collagen content and fiber type composition has been reported in livestock species [57]. Unfortunately, to date, proteomic studies related this topic in poultry are limited because of less practical importance in respect to meat tenderness issues in beef and pork meat.

## 4. Conclusions

It seems that main current problems related to meat quality in poultry are related to selection of the birds for growth rate and breast yield, even if involved underlying genetic mechanisms are not fully understood. This continues to create new challenges for the food scientists who must work with meat from birds selected primarily for quantitative traits. Today, with the advent of “omics” science there are more possibilities to further investigate these issues. Overall proteomics, even if is at an early stage, may allow the identification of markers for muscle growth and meat quality properties and understanding the molecular mechanisms that influence texture and water-holding capacity of meat.
